# The protean prion protein

**DOI:** 10.1371/journal.pbio.3000754

**Published:** 2020-06-25

**Authors:** Jesús R. Requena

**Affiliations:** CIMUS Biomedical Research Institute & Department of Medical Sciences, University of Santiago de Compostela-IDIS, Santiago de Compostela, Spain

## Abstract

The prion protein, PrP, can adopt at least 2 conformations, the overwhelmingly prevalent cellular conformation (PrP^C^) and the scrapie conformation (PrP^Sc^). PrP^C^ features a globular C-terminal domain containing 3 α-helices and a short β-sheet and a long flexible N-terminal tail whose exact conformation in vivo is not yet known and a metastable subdomain with β-strand propensity has been identified within it. The PrP^Sc^ conformation is very rare and has the characteristics of an amyloid. Furthermore, PrP^Sc^ is a prion, i.e., it is infectious. This involves 2 steps: (1) PrP^Sc^ can template PrP^C^ and coerce it to adopt the PrP^Sc^ conformation and (2) PrP^Sc^ can be transmitted between individuals, by oral, parenteral, and other routes and thus propagate as an infectious agent. However, this is a simplification: On the one hand, PrP^Sc^ is not a single conformation, but rather, a set of alternative similar but distinct conformations. Furthermore, other amyloid conformations of PrP exist with different biochemical and propagative properties. In this issue of *PLOS Biology*, Asante and colleagues describe the first murine model of familial human prion disease and demonstrate the emergence and propagation of 2 PrP amyloid conformers. Of these, one causes neurodegeneration, whereas the other does not. With its many conformers, PrP is a truly protean protein.

## Abstract 2

This Primer explores the implications of a study that describes the first murine model of familial human prion disease, demonstrating the emergence and propagation of two PrP amyloid conformers; of these, one causes neurodegeneration while the other does not. With its many conformers, PrP is a truly protean protein.

Prions were defined by Stanley Prusiner in 1982 as “proteinaceous infectious particles” [1]. This was likely a euphemism for “infectious protein,” a definition that would have been too explicit at the time. The first prion to be discovered was PrP^Sc^, identified as the causative agent of scrapie, a transmissible neurodegenerative disease of sheep [2]. Later, its normally folded precursor, PrP^C^, was found. Therefore, although PrP means “prion protein,” PrP^C^ is not a prion, rather, it can be refolded into a prion. Indeed, PrP^Sc^ prions propagate by templating their peculiar conformation into PrP^C^. This occurs through a process in which formation of hydrogen bonds between amino and carbonyl groups of the templating and templated polypeptides is likely to play a key role [3,4] (Fig 1). Prions are infectious because they can transmit from one individual to another, typically but not always (vide infra) by an oral route (Fig 1).

**Fig 1:**
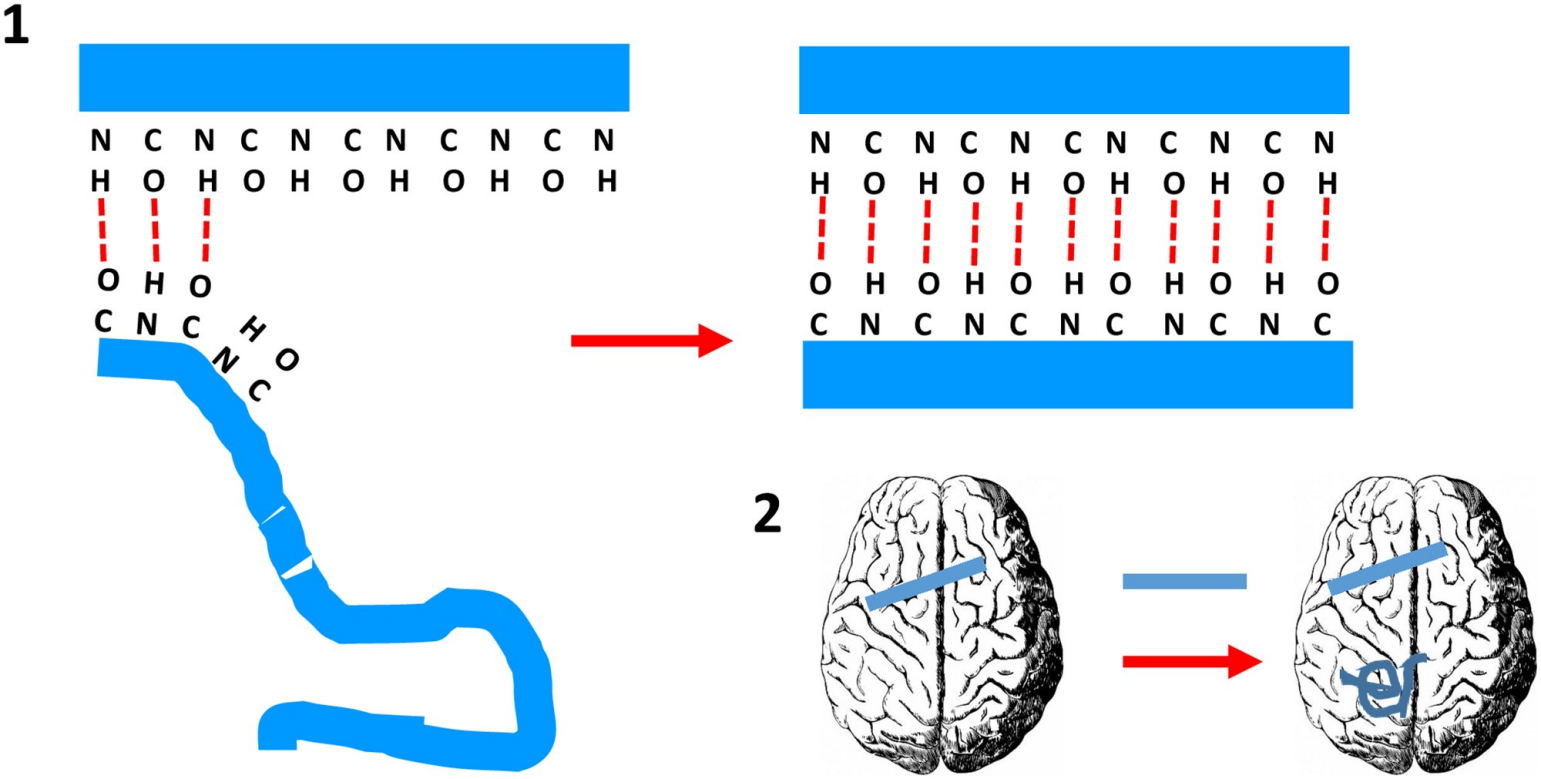
The propagative and infectious nature of prions. **(1)** Formation of hydrogen bonds between amino and carbonyl groups of the templating and templated polypeptides has been proposed as the key mechanism in prion propagation [3,4]. Carbonyl and amino groups in the edge β-strands of PrP^Sc^ are ready to form hydrogen bonds with an incoming, partially unfolded PrP polypeptide, coercing its refolding to form fresh β-strands. This way PrP^Sc^ can propagate throughout the brain. (**2)** Its infectious nature comes from the fact that PrP^Sc^, introduced in a different brain through oral, parenteral, or other means, can propagate there. PrP, prion protein; PrP^Sc^, prion protein with scrapie conformation.

Although at first sight it might appear so, prions do not contradict Anfinsen’s principle. The prion protein, encoded by the *Prnp* (human: *PRNP*) gene, time and again folds into a perfectly defined conformation, PrP^C^, featuring a globular C-terminal domain containing 3 α-helices, a short β-sheet (residues approximately 125–231), and a long flexible N-terminal tail (residues 23–124) [2]. The exact conformation of the tail in vivo is not yet known. A metastable subdomain with β-strand propensity has been identified within the 113–120 region [5]. It is only under rare circumstances that PrP^C^ refolds to adopt the alternative prion conformation, PrP^Sc^ (Fig 2). PrP^Sc^ is often an amyloid, and therefore, its conformation must allow stacking to form this kind of fibrillary structure [3]. Actually, adopting an alternative amyloid conformation is something that all proteins can do under certain circumstances, as demonstrated by Dobson and collaborators [6]. Any protein, no matter how well behaved and stable, if submitted to certain experimental conditions, such as low pH and/or presence of denaturants, will adopt an amyloid conformation [6]. In fact, the amyloid conformation is the most stable one, and all other native folds are believed to be kinetically trapped intermediate states [6]. Furthermore, all amyloids catalyze transition of their normal fold into the amyloid fold, a phenomenon known as “seeding” [7]. In summary, prions are just a special type of amyloids, and all proteins can be amyloids, so prions are not that strange.

**Fig 2:**
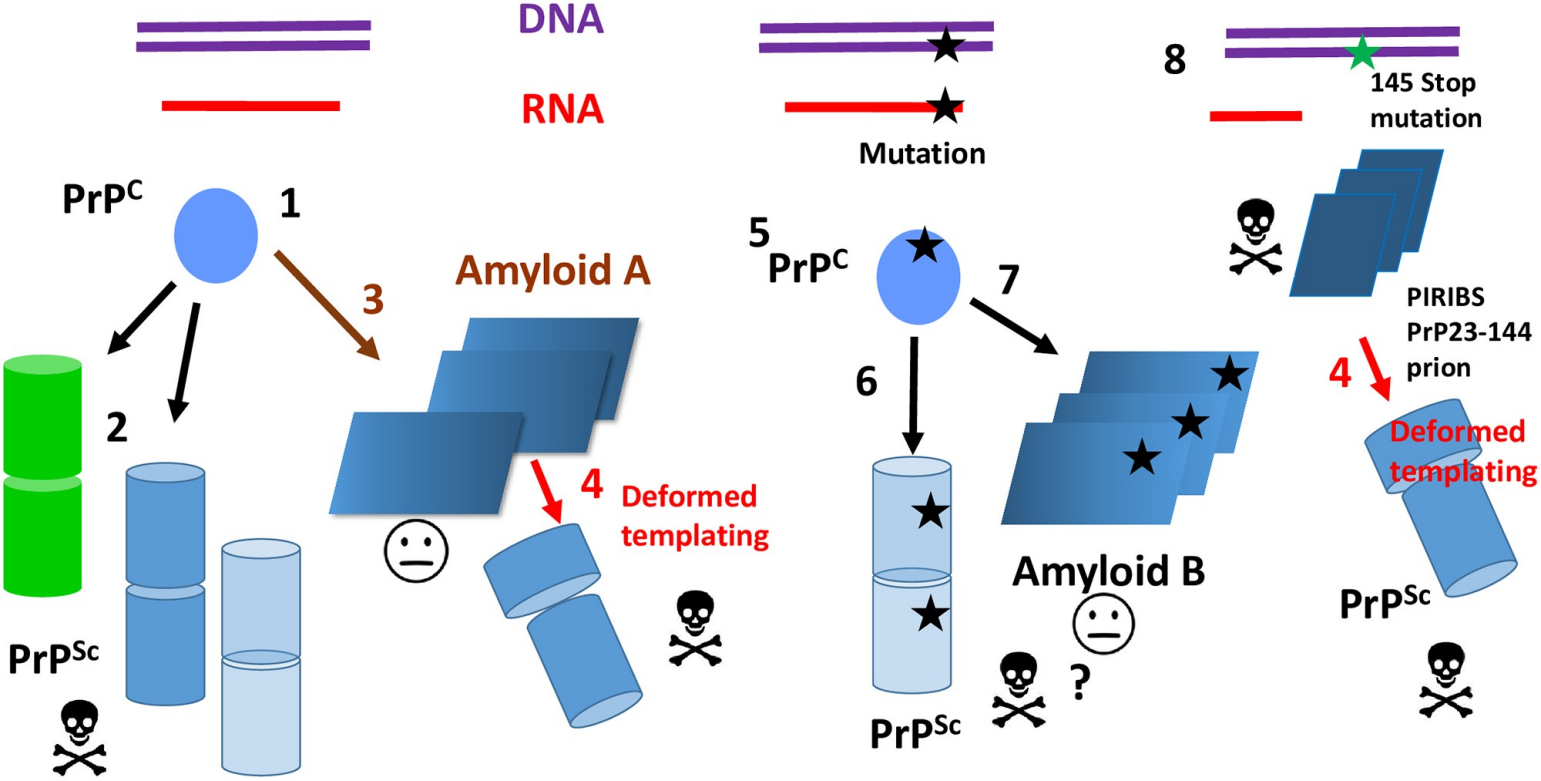
The many conformations of PrP. The *Prpn*/*PRPN* gene is transcribed and translated into the PrP polypeptide, which readily adopts the PrP^C^ conformation **(1)**. PrP^C^ can refold to adopt one of several PrP^Sc^ strain conformations **(2)**. These can be pathogenic (skull and bones sign) or not (face sign). PrP^Sc^ is depicted as stacked cylinders assuming that it is a 4Rβ [4], although its nature is not settled without doubt yet [25]. Different PrP^Sc^ strains exhibit different degrees of resistance to PK. PK-sensitive PrP^Sc^ exists (transparent cylinders). PrP^C^ can be also refolded in vitro and adopt a PIRIBS amyloid conformation that can propagate in the brain upon inoculation and can be further transmitted by inoculation as an infectious agent **(3)**. The darker shade indicates a PK-resistant C-terminus. During successive passages, deformed templating results in evolution of this conformer to PrP^Sc^ **(4)**. Different mutations in *PRNP* result in PrP^C^ **(5)** with a higher tendency to refold into a variety of propagative PrP conformers that form insoluble aggregates. In tg 117V mice, they include PK-sensitive, pathogenic PrP^Sc^ **(6)** and a transmissible, nonpathogenic PrP amyloid **(7)** with a characteristic pattern of resistance to PK (an approximately 8-kDa band corresponding to a segment that is different to that seen in the propagative amyloid described above and is signaled by the darker shading). It should be noted, however, that a recent study suggests that the approximately 8-kDa fragments are infectious and pathogenic and that they might exhibit a 2-rung solenoidal architecture [17]. A 145Stop mutation in *PRNP* results in PrP23-144 that folds into a propagative and pathogenic PIRIBS amyloid **(8)**. During passage to wild-type mice, deformed templating occurs, resulting in emergence of PrP^Sc^. PK, proteinase K; PIRIBS, parallel in register beta strand; PrP, prion protein; PrP^C^, prion protein with cellular conformation; PrP^Sc^ prion protein with scrapie conformation.

Prions do not contradict the Central Dogma of molecular biology either. In order to propagate, the prionic conformation PrP^Sc^ needs to recruit fresh PrP^C^ units and coerce them to refolding. Such PrP^C^ units are encoded by DNA transcribed to RNA and translated in ribosomes (Fig 2). Knock off the *Prnp* gene and there is no transmission of prions [2]. Again, prions are not that unusual, biologically speaking, contrary to their aura as obscure, bizarre proteins, acquired 25 years ago during the bovine spongiform encephalopathy (BSE) epizootic.

As I write this Primer at home, on day 30 of the severe acute respiratory syndrome coronavirus 2 (SARS-CoV-2) pandemic confinement, like millions around the world, it is inevitable to remember how about 20 years ago, everyone was anxiously looking at the curve of new cases of variant Creutzfeldt-Jakob disease (vCJD), the fatal neurodegenerative disease caused by bovine prions transmitted to humans [8]. At the time, it was not known whether bovine prions were very transmissible to humans or not. Virtually all Britons (with the exception of a few vegans) and countless other Europeans had been exposed to bovine prions, and considering that prion diseases are invariably fatal, fears of a disaster of apocalyptic proportions gripped epidemiologists and the general public [8]. Fortunately, bovine prions eventually proved to be very poorly transmissible to humans, and only approximately 200 deaths occurred, sad as all deaths are. Why is the transmission barrier between bovine prions and humans so high? We do not know. The sequences of bovine and human PrPs contain some amino acid differences, but how exactly these differences impinge in the templating process (Fig 1) to create a transmission barrier between bovine PrP^Sc^ of human PrP^C^ is not yet clearly understood.

Prion transmission barriers arising from differences in PrP sequence are not the only ones that exist. The classic studies performed by Bessen and Marsh showed the existence of 2 different kinds of prions in Syrian hamsters, both with the same sequence, that transmit differently. Thus, the Hyper PrP^Sc^ “strain” can be transmitted between hamsters by both intracerebral and intraperitoneal inoculation, whereas the Drowsy strain can be transmitted by intracerebral inoculation only [9]. Furthermore, these 2 strains exhibit distinct biochemical and biological properties. In fact, their names refer to phenotypic characteristics of hyperactivity or lethargy exhibited by infected animals. In an elegant study, Safar and colleagues showed that PrP^Sc^ strains must be subisoforms of PrP^Sc^, variations on a general structural theme [10]. This meant that there were not just 2 PrP conformers, PrP^C^ and PrP^Sc^: Rather, there were PrP^C^ and several relatively similar but distinct PrP^Sc^ conformers.

Once the BSE epizootic faded away (fears of a second wave of vCJD affecting more resistant individuals with longer incubation times have not materialized), attention was turned to sporadic prions. PrP^C^ sometimes refolds to PrP^Sc^ spontaneously, in the absence of any preexisting PrP^Sc^ template. It is a very rare event, with a yearly incidence of 1–2 cases/million people [2,8] and likely similar rates in other mammalian species. Once a small pool is formed, PrP^Sc^ prions propagate throughout the brain by templating. Sporadic human prions are, however, unable to infect another brain, unless they are taken there through specific and relatively uncommon events. These include ritual cannibalism (as in the case of kuru [2,8]), industrial cannibalism (as in the case of BSE [2,8]), and iatrogenesis (as in several instances of transmission through surgical instruments or treatment with brain-derived, prion-contaminated growth hormone [2,8]). In contrast, ovine scrapie and cervid chronic wasting disease (CWD) PrP^Sc^ prions are shed in feces and urine and are therefore readily transmitted between sheep and cervids. In fact, CWD and scrapie have become endemic in certain geographical regions [11]. But this only reflects differences in their physiology, not intrinsic differences in structure. In fact, sporadic human prions and “infectious” CWD and scrapie prions can be experimentally transmitted by inoculation with similar ease into appropriate transgenic (tg) mouse models [2,8].

Until recently, this was not the case, however, for familial prions. A number of mutations in the *PRNP* gene result in fatal neurodegenerative diseases whose phenotypes overlap with those of sporadic prion diseases [8]. In all familial cases, deposits of a PrP amyloid are found postmortem in the brain. It has therefore been assumed that these mutations predispose to conformational change in the expressed PrP protein, leading to the generation of disease-related PrP assemblies that propagate by seeded protein misfolding. Such propagative PrP assemblies, with amyloid characteristics, were therefore believed to be prions. Yet infectious transmission (i.e., transmission between brains) of familial pathogenic prions was not unequivocally achieved for a long time [8]. This might seem an oddity, of interest to punctilious specialists only. But it is not. First, if aggregates of mutant PrP were not infectious, were they prions? Could one be sure that at least, they propagated throughout the affected brain by seeded misfolding, or did they just misfold and clump in situ? And, if they were not infectious, was it because they display an additional conformation that is neither PrP^C^ nor any of the PrP^Sc^ subtypes?

But the picture was even more complicated. Mutant PrP aggregates could actually be transmitted by intracerebral inoculation. They were shown to propagate in the brain of recipient animals, which accumulated PrP amyloid deposits. Serial passage was also demonstrated. Yet these animals did not show signs of neurodegenerative disease [12,13]. This suggested that bona fide (i.e., infectious) prions could be innocuous. But then, what causes the brain damage seen in familial prion diseases? Yet another PrP conformer? Some unidentified pool of PrP^Sc^?

Asante and colleagues finally succeeded in experimentally transmitting a human familial disease. They showed that the aggregates of PrP with the A117V mutation, found in the brain of individuals suffering the deadly neurodegenerative Gerstmann-Sträussler-Scheinker (GSS) disease, could be transmitted to tg mice expressing human PrP on a mouse PrP null background, and along them, the disease [14]. This considerably simplified things. However, in order to minimize the transmission barrier associated to the A117V mutation, and therefore facilitate transmission, the recipient tg mice were engineered to carry the A117V mutation. Strikingly, these mice did not spontaneously develop a familial prion disease. This was very convenient: Had they developed the disease, it would have been impossible to assess the transmissibility of the HuPrP(A117V) aggregates present in the inoculum. But why did they not get spontaneously ill? Were they not producing prions in their brains?

In this issue of *PLOS Biology*, Asante and colleagues definitively close the circle by showing that some of the tg mice expressing human PrP 117V do spontaneously generate bona fide pathogenic prions [15]. Inoculation of abnormal PrP 117V assemblies found in their brains into other 117V tg mice produced, in some cases, a fatal neurodegenerative disease. The fact that the noninoculated mice do not develop the disease is therefore just a matter of timing: The longevity of mice is short, and the pathogenic prions accumulating in their brains do not have time to cause disease in all cases. The tg HuPrP117V mice can be considered a definitive mouse model of human familial prion disease.

Although the 2 studies by Asante and colleagues simplify our understanding of prion propagation, by allowing generalizations, they also bring fresh questions. The aggregates of mutant PrP 117V seen in affected brains show a very peculiar pattern of resistance to proteinase K (PK). PK has been used for many years as an important tool to characterize prions. Typically, PrP^Sc^ is partially resistant to PK, which trims its supposedly flexible N-terminal tail, generating a characteristic triplet of variably glycosylated resistant fragments termed PrP27-30. Small amounts of such triplet were seen in the infectious brain samples from PrP 117V tg mice, but only under certain circumstances, indicating that PrP^Sc^ exists in these brains but that it exhibits an unusually low resistance to PK [15] (Fig 2). The existence of PK-sensitive PrP^Sc^ has been known for a long time. Currently, it is not completely clear whether sensitivity to PK is a feature that depends on the tertiary or quaternary structure of a particular PrP^Sc^ strain.

Strikingly, these samples also contain noncanonical PK-resistant approximate 8-kDa fragments resulting from a double N- and C-terminal truncation. Similar fragments are detected in the brains of many prion diseases termed “atypical” [16]. The most parsimonious explanation for these fragments would be that they derive from a single PrP^Sc^ conformer. However, the interpretation of Asante and colleagues is that in their particular model, they come from 2 different PrP 117V conformers [14,15]. Among other considerations, the lack of correlation between accumulation of amyloid plaques in the brain and appearance of disease militates in favor of such interpretation. Thus, the doubly truncated fragment is proposed to derive from a transmissible but not infectious PrP amyloid conformer (Fig 2). It should be noted, however, that in a study published almost simultaneously to the one by Asante and colleagues, Vanni and colleagues inoculated GSS A117V brain homogenate to Bank voles (*Myodes glareolus*, a rodent that is very susceptible to prion infection), provoking a transmissible prion disease in them [17]. The brains of these animals also contained aggregates that upon treatment with PK yielded a doubly N- and C- truncated fragment. Then, Vanni and colleagues partially isolated the PK-resistant material and showed it to contain all the infectivity harbored in these brains [17]. These results strongly suggest that the infectivity in their model is associated with the PrP conformer that yields the approximately 8-kDa doubly truncated PK-resistant band. Although there seems to be a contradiction between the interpretations provided by these 2 groups, it should be noted that the models are different. It is particularly noteworthy that the infected Bank voles do not accumulate large amyloid deposits as the tgHuPrP117V do. In fact, electron microscopy images of the semipurified PK-treated infectious PrP material showed it not to be fibrillary, consisting of amorphous aggregates [17]. Further studies will be required to harmonize these fascinating results.

A propagative but noninfectious PrP amyloid isoform has been described by Baskakov and colleagues [18]. However, such amyloid yields C-terminal PK-resistant fragments, so it is not structurally identical to the one propagating among 117V tg mice. Furthermore, in successive passages, besides this PrP amyloid, bona fide PrP^Sc^ was seen to emerge with its characteristic PK-resistant triplet, and eventually, clinical disease appeared (Fig 2). This is the opposite of the results described by Asante and colleagues in which PK-sensitive PrP^Sc^ co-propagating along nonpathogenic PrP amyloid eventually faded away [15]. Baskakov and colleagues coined the term “deformed templating” to refer to the phenomenon by which their propagative amyloid slowly gives rise to PrP^Sc^ [18].

So the catalogue of PrP conformers has considerably expanded by now (Fig 2): There is PrP^C^; different versions (strains) of PrP^Sc^, some of which are resistant, whereas others are extremely sensitive to PK; and at least 2 distinct propagative PrP amyloids that can be serially transmitted between animals by inoculation, are not pathogenic, and generate distinctive patterns of PK-resistant fragments. Are these transmissible PrP amyloids prions? According to the original definition [1], yes, but they are hardly contagious at all: One must inoculate them intracerebrally to propagate them from brain to brain.

The structure of PrP^C^ is very well known [2]. But what about the other confomers? All of them are amyloids, but they exhibit very different biochemical and biological properties. The distinctive structural characteristic of amyloids is that the β-strands are stacked perpendicularly to the long axis of the amyloid. These β-strands are held together by an array of hydrogen bonds aligned with such axis. Currently, there are only 2 structural amyloid models relevant to propagative PrP: the parallel in register beta strand (PIRIBS) structure and the 4-rung β-solenoid (4RβS). In the PIRIBS structure, each PrP molecule is a flat, serpent-like structure. Different PrP molecules stack on top of each other “in register,” this is, each amino acid residue is exactly on top of the equivalent residue in the preceding PrP monomer [19]. Solid-state NMR and electron paramagnetic resonance (EPR) spectroscopy data strongly suggest that the nonpathogenic, propagative amyloid described by Baskakov and collaborators features a PIRIBS architecture [20]. Smaller PrP fragments are also known to fold into PIRIBS structures [21]. Another propagative (but this time pathogenic) PIRIBS amyloid comprising PrP23-144 subunits has been described by Surewicz and colleagues [22]. It should be considered a bona fide PIRIBS PrP prion, but because it comprises a truncated version of PrP, it must be excluded from the catalogue of full-length PrP conformers. However, it is mentioned here for 2 reasons: On the one hand, an amber mutation of PrP (PrP145Stop) exists that results in expression of truncated PrP23-144 and leads to a familial prion disease. On the other, the characteristic N- and C-truncated fragment, resulting from PK treatment of A117 propagative, nonpathogenic PrP amyloid, involves a similar C-truncation, so it is tempting to speculate that both amyloids share a similar architecture (Fig 2). It should be noted that during successive passage of PrP23-144 prions in wild-type mice, classic PrP^Sc^ also emerges, as in the cases described by Baskakov and colleagues. [18]. It is not clear to what degree each conformer of PrP contributes to pathogenesis. At this point, it is worth mentioning that some authors have suggested that even in simpler cases of prion disease such as sporadic ones, PrP^Sc^ is not the pathogenic conformer: Rather, a “toxic” species exists that derives from PrP^Sc^ [23]. Yet another possible conformer, whose discussion lies outside of the scope of this primer.

In the proposed 4RβS structure, PrP coils around itself, and β-strands (3 in each one of the 4 rungs) are on top of other β-strands of different sequence, and therefore, stacking is not in register [4]. Besides agreeing with cryo-EM microscopy and fiber X-ray diffraction data, a 4RβS atomic model is the only currently available model that is physically plausible, i.e., it stays stable through molecular dynamics simulations [4]. It also allows substantial room to accommodate the bulky glycans of stacked PrP^Sc^ subunits [4]. Preliminary solid-state NMR (ssNMR) studies of recombinant PrP^Sc^ are fully compatible with this model [24], but in the absence of more conclusive data, the nature of the structure of PrP^Sc^ remains still unsettled [25].

The term “protein” was introduced by the Dutch chemist Gerardus Johannes Mulder in 1838, following a suggestion by Jöns Jakob Berzelius, in his classic paper on the composition of some animal substances [26]. He derived the word from the Greek term *protos* (*πρῶτος*), meaning primordial, first. This term is also in the origin of the name of the god Proteus, perhaps because he was the first son of Poseidon. The main characteristic of Proteus was his metamorphic ability to change shape and appearance. The etymologic coincidence is very becoming, given that proteins exhibit an enormous variety of forms and functions. However, each protein typically has one form—not PrP, a truly protean protein, with many conformations, all of them with peculiar properties. The prion concept, the idea that a protein conformation could be infectious (i.e., capable of propagating between individuals, irrespective of whether it causes damage or not) was initially met with skepticism. Yet it seems that PrP can generate not 1, but perhaps as many as 3 prions, 4 if it is truncated. Prions may defy neither the Central Dogma of molecular biology nor Anfinsen´s principle, but perhaps it could be said that the protean array of PrP conformations defy Occham’s razor.
